# S100 family proteins are linked to organoid morphology and EMT in pancreatic cancer

**DOI:** 10.1038/s41418-023-01126-z

**Published:** 2023-02-24

**Authors:** Ronnie Ren Jie Low, Ka Yee Fung, Hugh Gao, Adele Preaudet, Laura F. Dagley, Jumana Yousef, Belinda Lee, Samantha J. Emery-Corbin, Paul M. Nguyen, Rune H. Larsen, Nadia J. Kershaw, Antony W. Burgess, Peter Gibbs, Frédéric Hollande, Michael D. W. Griffin, Sean M. Grimmond, Tracy L. Putoczki

**Affiliations:** 1 The Walter and Eliza Hall Institute of Medical Research, Parkville, VIC 3052 Australia; 2 Department of Medical Biology, The University of Melbourne, Parkville, VIC 3052 Australia; 3 University of Melbourne Centre for Cancer Research, Victorian Comprehensive Cancer Centre, Parkville, VIC 3000 Australia; 4 Department of Clinical Pathology, University of Melbourne, Parkville, VIC 3000 Australia; 5 Centre for Cancer Research, Hudson Institute of Medical Research, Clayton, VIC 3168 Australia; 6 Department of Molecular and Translational Science, Monash University, Clayton, VIC 3800 Australia; 7 Department of Biochemistry and Pharmacology, Bio21 Molecular Science and Biotechnology Institute, University of Melbourne, Parkville, VIC 3000 Australia

**Keywords:** Cancer models, Cancer microenvironment

## Abstract

Epithelial-mesenchymal transition (EMT) is a continuum that includes epithelial, partial EMT, and mesenchymal states, each of which is associated with cancer progression, invasive capabilities, and ultimately, metastasis. We used a lineage-traced sporadic model of pancreatic cancer to generate a murine organoid biobank from primary and secondary tumors, including sublines that underwent partial EMT and complete EMT. Using an unbiased proteomics approach, we found that organoid morphology predicts the EMT state, and the solid organoids are associated with a partial EMT signature. We also observed that exogenous TGFβ1 induces solid organoid morphology that is associated with changes in the S100 family, complete EMT, and the formation of high-grade tumors. S100A4 may be a useful biomarker for predicting EMT state, disease progression, and outcome in patients with pancreatic cancer.

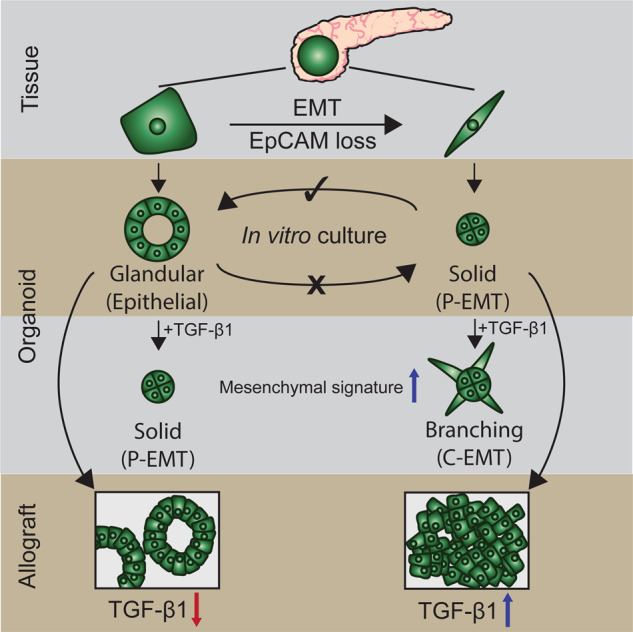

## Introduction

Pancreatic ductal adenocarcinoma (PDAC) is among the most lethal malignancies, with a 5-year survival rate of less than 12% [1]. Many patients succumb to the disease within the first 6 months, reflecting late diagnosis, metastasis, and therapy resistance [2, 3]. A distinctive feature of PDAC is the progression of early pancreatic intraepithelial neoplasia to an advanced unresectable disease [2, 3]. During this process, the histopathological phenotype of the tumor changes from low-grade, characterized by well-formed glandular epithelial structures, to high-grade, whereby epithelial cells are diffuse throughout the desmoplastic stroma [2, 3]. This process of dissemination has been linked to the activation of epithelial-mesenchymal transition (EMT), a cell-biological program that is essential for the early stages of embryogenesis and organ formation and is exploited by cancer cells [4]. EMT supports pathological events associated with the loss of epithelial behavior and the acquisition of mesenchymal features that enable invasion and metastasis [4].

In PDAC, patient analysis, in vitro assays, xenograft models, and genetically engineered mouse models (GEMMs) have suggested that a set of transcription factors (TF) enables EMT [5–10]. We now know that PDAC progression is not solely driven by the classic EMT-TFs Snail or Twist [6]; however, the loss of EMT-TF Zeb1 can result in the growth of low-grade tumors [8]. These EMT-TFs were originally considered part of a binary process, whereby cells transition into two distinct epithelial or mesenchymal states, the latter defined by the loss of expression of the epithelial protein E-cadherin and gain of expression of the mesenchymal protein vimentin [4]. This concept has now shifted to reflect a continuum of partial EMT states with overlapping transcriptional features found in multiple cancers, including PDAC [4, 11]. Cancer cells can also revert from partial EMT to epithelial states through mesenchymal-epithelial transition (MET) [9, 10]. This spectrum of heterogeneous EMT states may be associated with differences in epithelial plasticity and cell migration, thereby influencing tumor progression, metastasis, and response to treatment [11, 12]. The intrinsic mechanisms that drive the partial EMT or complete EMT remain unclear.

PDAC tumor cells reside within a dense desmoplastic stroma comprising cancer-associated fibroblasts (CAFs) and associated extracellular matrix (ECM), which constitutes more than 80% of the tumor mass [13]. The mechanism by which the stroma modulates the behavior of tumor cells, particularly the progression of EMT, remains unclear. A primary focus has been placed on the cytokine TGFβ1, which is potently secreted by CAFs [14], resulting in autocrine signaling and differentiation into myofibroblastic CAFs that promote the deposition of ECM proteins [15, 16]. Paracrine TGFβ1 signaling can also act on benign neoplastic epithelial cells, leading to cell cycle arrest or on neoplastic cells, to induce proliferation, motility, and EMT [17–21]. How stromal-derived and cell-intrinsic mechanisms converge on EMT phenotypes in cancer cells is unclear, and the identification of transitions between different stages of the EMT continuum is technically challenging.

Here, we used an unbiased quantitative proteomics approach to explore the relationship between murine organoid morphology, the EMT continuum, and tumor grade. In a murine model of PDAC, organoids with solid morphology underwent partial EMT and formed high-grade tumors when transplanted into syngeneic hosts. We further explored the effects of contextual signals from cytokines derived from the stromal microenvironment on EMT induction. We demonstrated that TGFβ1 can induce changes in organoid morphology and provide evidence that this is associated with the induction of complete EMT and alterations in S100 family expression. Collectively, our findings suggest that S100A4 may be a useful biomarker for predicting EMT state, disease progression, and survival.

## Results

### Murine organoids have two distinct morphological subtypes

**Fig. 1:**
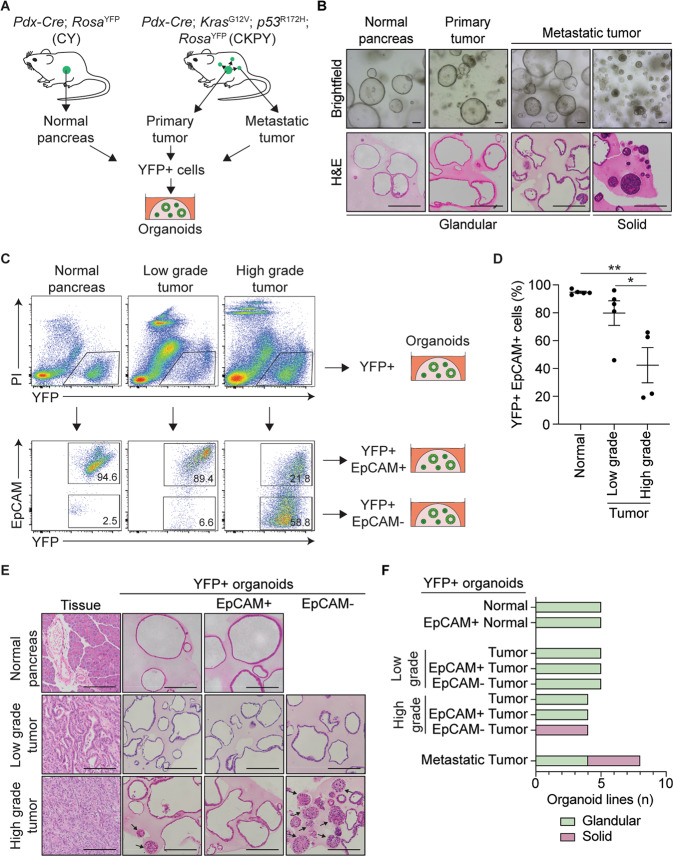
Generation of a murine organoid biobank with different morphological features. **A** Schematic representation of the workflow to generate organoids from primary and metastatic secondary tumors in the KPC model. YFP + cells FACS isolated from *Pdx-Cre*; *Rosa*^YFP^ (CY) mice were used to generate normal pancreatic organoids, while YFP + cells FACS isolated from either primary tumors or secondary tumors (liver, lung, and diaphragm) from *Pdx-Cre*; *Kras*^G12V^; *p53*^*R*172H^; *Rosa*^YFP^ (CKPY) mice were used to generate tumor organoids. **B** Representative brightfield (top) and H&E (bottom) images of normal pancreatic, primary tumor and metastatic tumor organoids. Representative glandular and solid subtype organoids are shown. Scale bar = 100 μm. **C** Representative FACS plots for isolation of YFP + EpCAM+ and YFP + EpCAM- cells from normal pancreas (*N* = 5), low-grade tumor (*N* = 5) and high-grade tumor (*N* = 4) for the generation of organoids. **D** Percentage of live YFP + EpCAM+ cells isolated from the normal pancreas from CY mice (*N* = 5), and either low-grade tumors (*N* = 5) or high-grade (*N* = 4) pancreatic tumors from CKPY mice. Each dot represents an individual mouse. Data are presented as mean + /− SEM. *p < 0.05, Student’s *t*-test. **E** Representative H&E images of the primary tumor and corresponding YFP + (left), YFP + EpCAM + (middle) and YFP + EpCAM- (right) organoids. Scale bar = 100 μm. **F** Quantification of the organoid morphology for each MO line, represented as glandular (green) or solid (red).

*Pdx*^Cre^, *Kras*^G12V^, *p53*^*R*172H^, *Rosa*^YFP^ (CKPY) GEMM of PDAC were used to generate a murine organoid biobank, as the YFP allele permitted identification (Fig. S1A, B) and FACs isolation (Fig. 1A) of metastatic lesions in the lungs, liver, and diaphragm (Table S1). Pdx-1 drives cre-recombinase in pancreatic epithelial cells allowing constitutive expression of a transition mutation, LSL-*Kras*^G12D^, that activates the Ras effector pathway and a point mutation in the tumor suppressor, p53 (LSL-*Trp53*^R172H^), that functionally equivalent to a dominant-negative mutation. Littermate *Pdx*^Cre^; *Rosa*^YFP^ (CY) mice were used to isolate epithelial cells from healthy pancreata (Fig. 1A; Table S1). On average, approximately 13% of the cells detected in CY pancreas were YFP + epithelial cells, with no YFP + cells detected in the liver, diaphragm, or lungs, as expected (Fig. S1C, D). In CKPY mice, on average, approximately 17% of the cells detected in the pancreas were YFP + epithelial cells, and YFP + cells were also detected in the liver, diaphragm, lung, and blood, although the percentage of YFP + cells detected in these organs varied between the mice (Fig. S1D). There was a 100% success rate for the generation of normal pancreatic and pancreatic tumor organoids, whereas the success rates for the generation of organoids from metastatic lesions varied (Fig. S2A, B).

We observed two distinct organoid morphological subtypes: glandular and solid. H&E staining demonstrated that the glandular organoid subtype retained a single cellular layer with a central lumen, whereas the solid organoid subtype often had multiple cellular layers in the absence of a lumen (Fig. 1B). There was no correlation between the tissue origin and morphological subtypes (Fig. S2A). We investigated whether the differences in morphology observed were reflective of different states of EMT, following the observation that CKPY mice with high-grade tumors often displayed regions with loss of expression of the epithelial marker EpCAM, whereas the same epithelial cells expressed the mesenchymal marker vimentin, consistent with the classic EMT phenotype (Fig. S1B) [5]. Similar to other studies, we found that the loss of E-cadherin was not as consistent as the loss of EpCAM staining when examining EMT phenotypes [11, 22] (Fig. S1B).

In parallel with FACS sorting of primary tumors for YFP + cells, YFP + EpCAM+ and YFP + EpCAM- cells were also sorted to generate partial EMT organoid sublines (Fig. 1C). We found that fewer YFP + EpCAM+ cells were present in the pancreas of mice that were retrospectively assigned to high-grade tumors by histopathology, which is consistent with the suggestion that some malignant cells had undergone EMT (Fig. 1D). All the YFP + EpCAM+ cells that were sorted from the normal pancreas of CY mice successfully formed organoids; however, the low percentage of YFP + EpCAM- cells isolated did not grow (Fig. 1E; Fig. S2B). Within the established organoids, the YFP + EpCAM+ and matched YFP + normal pancreatic organoids retained a glandular morphology across passages (Fig. 1E, F, Fig. S2C). All organoids established from YFP + EpCAM+ and YFP + EpCAM- cells from the pancreatic tumors of CKPY mice grew successfully (Fig. 1E, F; Fig. S2C). We noticed that low-grade primary tumors resulted in a higher percentage of glandular subtype organoids in YFP + organoids, whereas high-grade tumors generated a mixture of solid and glandular subtype organoids (Fig. 1E, F; Fig. S2C). For the YFP + EpCAM+ and YFP + EpCAM- organoid sublines, low-grade tumors generated glandular organoids (Fig. 1E, F; Fig. S2C). Although YFP + EpCAM- MO from low-grade tumors was initially a solid subtype, it transitioned to a glandular subtype after serial passaging (Fig. S2C), suggesting that the cells had undergone MET in culture. In contrast, YFP + EpCAM- organoids from high-grade tumors retained their solid morphology, suggesting differences in the plasticity of organoids generated from either low- or high-grade tumors (Fig. S2C).

### Murine organoid morphology is linked to elevated s100a4 and decreased s100a14 expression

**Fig. 2:**
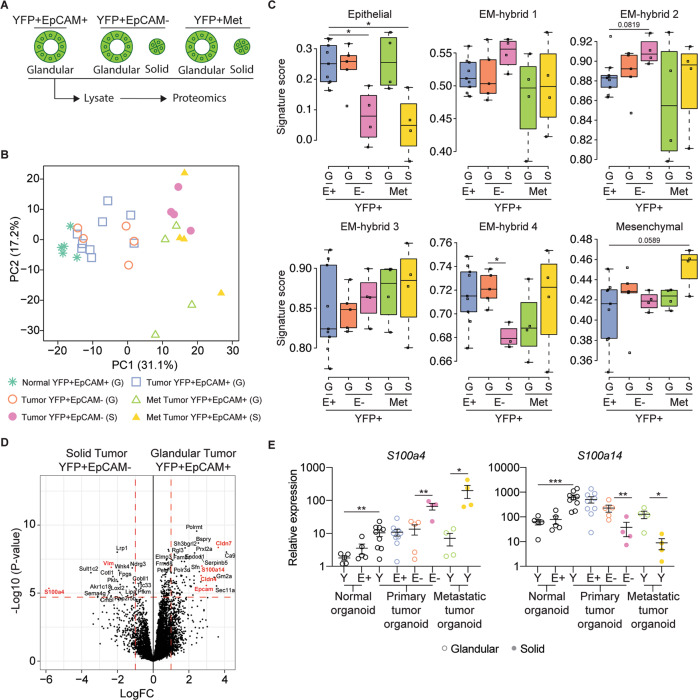
Murine organoid morphology correlates with partial EMT state. **A** Schematic representation of organoid lines used for label-free based quantitative proteomics analysis. Organoids with different morphologies were subjected to cell lysis followed by on-bead enzymatic digestion and subsequent mass spectrometry analysis. **B** Principal component analysis plot of 3546 of the most variable proteins across organoid lines, including YFP + EpCAM+ normal (*N* = 5), YFP + EpCAM+ tumor (*N* = 9), YFP + EpCAM- tumor (*N* = 9) and YFP + secondary tumors (Met; *N* = 8) organoids. The plot shows the separation of samples based on different principal components (PCs). Glandular (G) and solid (S) organoid lines are indicated. **C** The organoid signature score of the six previously described EMT subtypes [22] including YFP + EpCAM + (E+; *N* = 9), YFP + EpCAM- (E-; *N* = 9) and YFP + secondary (Met; *N* = 8) tumor organoids. Glandular (G) and solid (S) organoid lines are indicated. **D** Volcano plot illustrating the log_2_ protein ratios in whole cell lysates of organoids, comparing glandular YFP + EpCAM+ with solid YFP + EpCAM- tumor organoids. Proteins were deemed differentially regulated in the log2 fold change in protein expression was ≥ 1-fold and exhibited an adjusted *p* ≤ 0.05. **E** mRNA expression of *S100a4* and *S100a14* in YFP + (Y), YFP + EpCAM + (E + ) and YFP + EpCAM- (E-) organoids. Each dot represents an individual organoid line. Glandular (open circle) and solid (closed circle) organoid lines are indicated. Data is relative to *Gapdh*, presented as mean + /− SEM. *p < 0.05; **p < 0.01; ***p < 0.001, Mann–Whitney test.

To determine whether the morphology of the organoids was linked to the EMT continuum, we performed label-free quantitative proteomic analysis of lysates generated from the YFP + EpCAM+ primary tumors, YFP + EPCAM- primary tumors, and YFP + secondary tumor organoids (Fig. 2A; Table S2). The principal component analysis plot was generated using a singular value decomposition approach. Information related to data variability, correlations between variables and principal components were calculated based on log intensities of 3546 proteins that were identified across all organoid lines including YFP + EpCAM+ normal (*N* = 5), YFP + EpCAM+ tumor (*N* = 9), YFP + EpCAM- tumor (*N* = 9) and YFP + secondary tumor (Met; *N* = 8) organoids. PCA revealed a clear pattern of separation between the different organoid sublines including glandular (G) and solid (S) morphologies (Fig. 2B). Next, we generated an EMT score based on a previously defined continuum of partial EMT signatures, namely epithelial, EM-hybrid (H1-4), and mesenchymal [22]. As YFP + EpCAM+ epithelial cells were utilized to generate organoids these were used as a baseline for comparison of EMT signatures. The glandular primary tumor YFP + EpCAM+ and glandular metastatic YFP + organoids showed no clear association with an EM-hybrid or mesenchymal signature but maintained an epithelial signature (Fig. 2C). The glandular YFP + EpCAM- organoid generated from low-grade primary tumors also had an enriched epithelial signature (Fig. 2C). We found that solid YFP + EpCAM- and solid YFP + metastatic organoids had a lower epithelial signature, consistent with their entry into the classic EMT state (Fig. 2C). We also observed a lower H4 signature in the solid primary tumor YFP + EpCAM, indicating partial EMT, whereas the solid YFP + metastatic organoids had a higher mesenchymal signature, suggesting that the metastatic organoids progressed towards complete EMT (Fig. 2C).

We were curious whether our proteomics dataset could identify individual proteins associated with morphological changes and the EMT status. To this end, we first examined the differential expression between normal glandular YFP + EpCAM+ and glandular tumor YFP + EpCAM+ organoids (Fig. S3A) and found an elevation of Agr2 in tumor organoids, a protein in the TP53 pathway that has previously been shown to be expressed in human PDAC patients and is linked to tumor cell dissemination [23]. Other differentially expressed proteins included Nusap1, a tubulin protein involved in cell cycle regulation, and Lgals4, a carbohydrate-binding protein (Fig. S3A), both of which are associated with a poor prognosis in patients with PDAC [24, 25]. Examination of differential protein expression between the glandular primary tumor organoid partial EMT sublines revealed no major differences in protein expression (Fig. S3B), whereas the glandular metastatic tumor organoid had reduced Cldn7 compared to the glandular primary tumor organoids (Fig. S3C).

Next, we compared the ‘epithelial’ glandular YFP + EpCAM+ and solid YFP + EpCAM- organoid sublines, which showed increased EpCAM expression in the primary tumor glandular organoids and increased vimentin expression in the solid organoids, consistent with their partial EMT phenotypes (Fig. 2D). In solid organoids, there was also a significant reduction in the expression of Cldn7 and Cldn4, which belong to the tight junction protein family previously associated with tumor differentiation, liver metastasis, and decreased survival in patients with PDAC [26, 27]. In contrast, solid metastatic organoids showed a reduction in EpCAM and Cldn7 levels compared to glandular primary tumor organoids (Fig. S3D). Comparison of the solid and glandular YFP + EpCAM- sublines also revealed decreased expression of Cldn7 and EpCAM in the solid organoid (Fig. S3E), whereas comparisons of organoids from metastases did not reveal major differences from the solid organoids (Figs. S3F–H). An overall comparison of the top 25 differentially expressed proteins (Fig. 2D) within the MO sublines demonstrated that the expression profile of organoid was similar within morphological subtypes (Fig. S3I).

We were intrigued by the differential expression of S100a4 and S100a14 in our dataset (Fig. S3I–K). The S100 family of proteins was recently found to be among the most abundant secreted factors in PDAC when compared with the normal pancreas in both murine models and patients [28]. We observed the differential expression of S100a4, S100a13, S100a14, and S100a16 between glandular YFP + EpCAM+ and solid YFP + EpCAM − organoids (Fig. 2D; Fig. S3J). Further examination of *S100a4* mRNA expression levels confirmed increased expression in YFP + tumor organoids compared to that in YFP + normal organoids, with a further significant increase in solid organoids (Fig. 2E). In contrast, while *S100a14* mRNA was significantly increased in YFP + tumor organoids compared to YFP + normal organoids, its expression was decreased in solid organoids (Fig. 2E). Publicly available transcriptomic data from patients with pancreatic cancer and normal human pancreatic tissue also confirmed increased expression of both *S100A4* and *S100A14* in PDAC (Fig. S4B) [29]. We investigated whether S100 proteins were associated with tumor grade, examined publicly available transcriptomic data from pancreatic cancer patients with high tumor purity [30], and confirmed the association of *S100A4* with high-grade tumors and *S100A14* expression with low-grade tumors (Supplementary Fig. S3K).

### TGFβ1 induced solid morphology is associated with increased s100a4 expression

TGFβ1 has previously been associated with EMT [18, 20]. In patients, the TGFβ1 signature was significantly elevated in high-grade tumors (Fig. S4C). In CKPY mice, we also observed that *tgfb1*, along with its receptor *tgfbr2*, increased in high-grade murine tumors (Fig. S4D). However, within the partial EMT organoid sub-lines, we did not observe a significant increase in the expression of endogenous *tgfb1*, but observed a trend of decreased expression along with a significant reduction in *tgfbr2* expression in solid organoids (Fig. S4E).

**Fig. 3:**
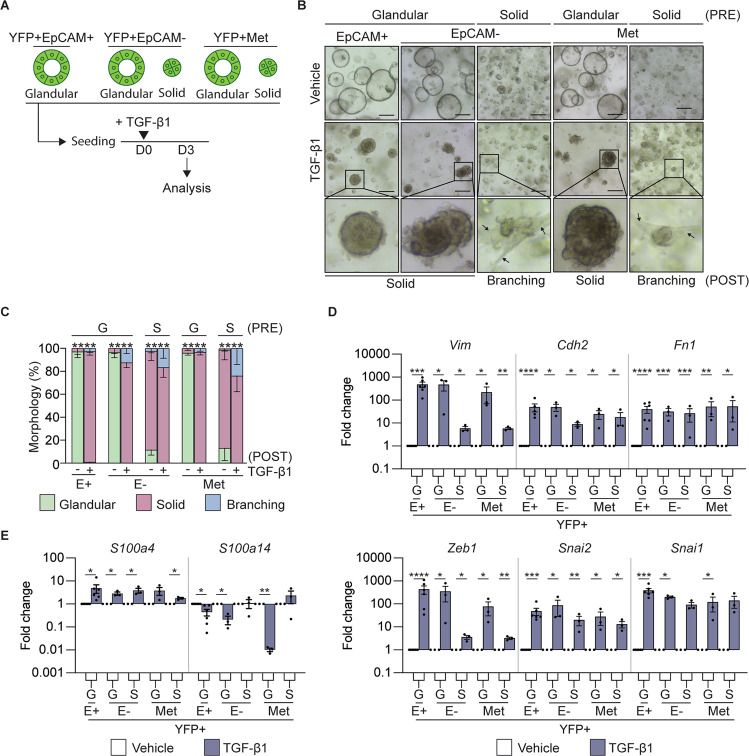
TGF-β1 treatment induces a mesenchymal signature in tumor organoids. **A** Schematic representation of the treatment of murine tumor organoids seeded as single cells with recombinant TGFβ1 and timing of quantification of the morphology and gene expression signatures. **B** Representative brightfield images of YFP + EpCAM + (*N* = 6), YFP + EpCAM- (*N* = 6) and YFP + secondary (Met, *N* = 6) tumor organoids on day 3 post TGFβ1 treatment. The pre-treatment (PRE) organoid morphology is indicated. Representative images of organoids post-treatment (POST) are shown. Arrows indicate a branching phenotype. Images are representative of 3 biological replicates. Scale bar = 300 μm. **C** Quantification of the tumor organoid morphology 3 days post TGFβ1 treatment. YFP + EpCAM + (*N* = 6), YFP + EpCAM- (*N* = 6) and YFP + secondary (Met; *N* = 6) tumor organoids. Organoids are grouped as glandular (G) and solid (S) morphologies pre-treatment (PRE). Quantification of glandular (green), solid (red) and branching (blue) post cytokine treatment (POST). Data includes 3 biological replicates and is presented + /− SEM. ****p < 0.0001, Chi-square test. **D**, **E** mRNA expression levels of mesenchymal markers, *Vim*, *Cdh2*, *Fn1*, *Snai1*, *Snai2*, *Zeb1* (**D**); and S100 family members, S*100a4*, *S100a14* (**E**), in YFP + EpCAM + (E+), YFP + EpCAM- (E-) primary tumor organoids and YFP + secondary tumor organoids following the addition of TGFβ1 (blue). Each dot represents an individual organoid line. Organoids are grouped as glandular (G) and solid (S) morphologies. Data includes three biological replicates and is presented as log10 fold change relative to the vehicle (white) control, mean + /− SEM. *p < 0.05, **p < 0.01, ***p < 0.001, ****p < 0.0001, paired *t*-test.

Since the contribution of TGFβ1 to EMT may be contextual, we mimicked the high levels of TGFβ1 secreted by the tumor microenvironment [31, 32] in vitro through treatment of single cell seeded organoid cultures with recombinant TGFβ1 (Fig. 3A). We observed a reduction in the number of organoids that formed in the TGFβ1 groups, that was not related to the morphology of the organoid (Fig. S4F) and could be related to TGFβ1 induced cell death [33]. However, we observed that glandular organoids adopted a solid morphology following TGFβ1 treatment, while a portion of the solid organoids began to display an invasive ‘branching’ phenotype (Fig. 3B, C). The organoids were harvested at the indicated end point and unbiased proteomics (Fig. S4G, H) or gene expression analysis by qRT-PCR (Fig. 3D) demonstrated that these TGFβ1 induced morphological changes were associated with a changes in the expression of classic mesenchymal markers, including *Zeb1, Snai1/2, Vim, Cdh2 and Fn1*, suggesting induction of a partial EMT phenotype. We also observed a significant increase in the expression of *S100a4* and a significant decrease in the expression of *S100a14* (Fig. 3E), which regulated the expression of these genes downstream of the TGFβ1 pathway.

**Fig. 4:**
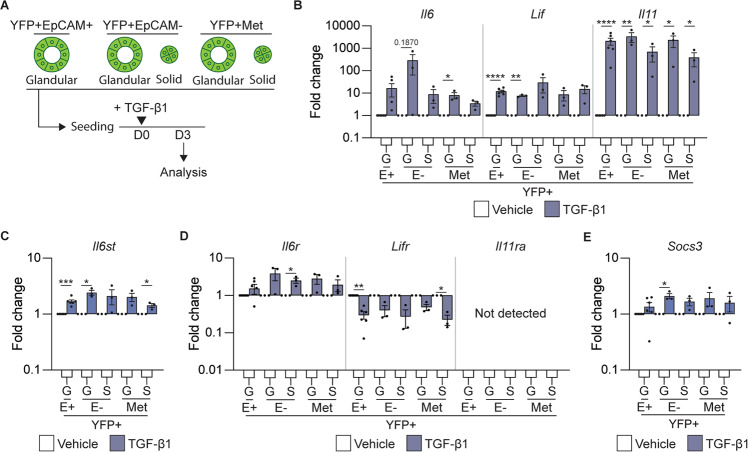
TGFβ1 induces the expression of IL-6 family cytokines. **A** Schematic representation of the treatment of murine tumor organoids seeded as single cells with recombinant TGFβ1 and the timing of quantification of gene expression signatures. **B**–**E** mRNA expression levels of IL-6 family cytokines *Il6*, *Lif*, and *Il11* (**B**); IL-6 family cytokine receptors *il6st* (**C**); *Il6r*, *Lifr*, and *Il11r* (**D**), and the IL-6 cytokine family target gene, *Socs3* (**E**), 3 days post treatment with TGFβ1. Each dot represents an individual organoid. Organoids are grouped into glandular (G) and solid (S) types. Data includes three biological replicates and is presented as log10 fold change relative to the vehicle (white) control (mean ± SEM). *p < 0.05, **p < 0.01, ***p < 0.001, ****p < 0.0001, paired *t*-test.

Given the adverse outcomes observed following direct targeting of TGFβ1 in pancreatic cancer patients [34], we explored whether other cytokines downstream of TGFβ1, which may be more favorable therapeutic targets, were implicated in the EMT-associated changes in morphology that we observed. To this end, we confirmed that following treatment of organoid cultures with recombinant TGFβ1 (Fig. 4A), the expression of the IL-6 family cytokines *il6*, *lif,* and *il11* was significantly increased (Fig. 4B). Interestingly, each of these cytokines or their receptors has been implicated in EMT [35, 36]. In publicly available transcriptomic datasets, *IL6* was associated with high-grade tumors in patients and *LIF* was associated with low-grade tumors (Fig. S5A). We hypothesized that autocrine IL-6 family cytokine signaling would be augmented in TGFβ1 treated organoids, as we also observed increased expression of the shared signaling receptor *il6st* (gp130; Fig. 4C). We were curious as to whether a hierarchy of importance exists within this cytokine family, as we observed increased expression of the cognate cytokine receptor, *il6ra* and decreased expression of *lifr* in TGFβ1 treated organoids (Fig. 4D). We did not observe *il11ra* in tumor organoids (Fig. 4D), contrary to previously published reports [37]. Consistent with the activation of an autocrine IL-6 signaling cascade, *Socs3*, a STAT3 target gene that acts as a negative physiological regulator of signaling, also increased following stimulation with recombinant TGFβ1 (Fig. 4E).

**Fig. 5:**
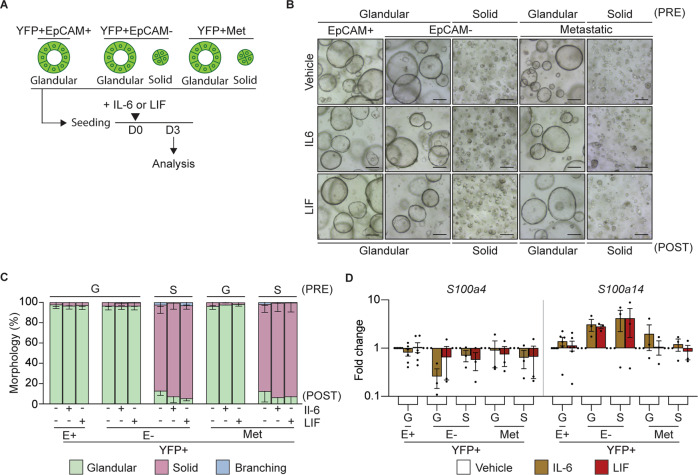
IL-6 or LIF do not alter tumor organoid morphology or induce EMT. **A** Schematic representation of the treatment of murine organoids seeded as single cells with recombinant IL-6 or LIF and timing of quantification of the morphology and gene expression signatures. **B** Representative brightfield images of YFP + EpCAM + (E+; *N* = 6), YFP + EpCAM- (E-) and YFP + secondary (Met; *N* = 12) tumor organoids on day 3 post treatment (POST) with the indicated cytokine. Organoids are grouped as glandular (G) and solid (S) morphologies pre-treatment (PRE). Scale bar = 300 μm. **C** Quantification of the tumor organoid morphology 3 days post treatment with the indicated cytokine. YFP + EpCAM + (E + , *N* = 6), YFP + EpCAM- (E-, *N* = 12 primary and secondary). Glandular (green), solid (red) and branching (blue). Data is presented + /− SEM. Results are not significant, Chi-square test. **D** mRNA expression levels of S100 family proteins, S*100a4* and *S100a14*, in YFP + EpCAM + (E+), YFP + EpCAM- (E-) primary tumor organoids and YFP + secondary tumor organoids following the addition of the indicated cytokine (IL-6, brown; LIF, red). Each dot represents an individual organoid. Organoids are grouped as glandular (G) and solid (S) morphologies. Data includes 3 biological replicates and is presented as log10 fold change relative to the vehicle (white) control, mean + /− SEM. paired *t*-test.

To determine whether IL-6 and LIF directly induced EMT, we cultured organoids in the presence of either recombinant IL-6 or recombinant LIF (Fig. 5A); however, we did not observe a change in organoid morphology (Fig. 5B, C). Consistent with the lack of EMT induction, we did not observe an increase in the expression of the classic mesenchymal markers (Fig. S5B) and *s100a4*, we observed an increase in *s100a14* (Fig. 5D), which has previously been associated with IL-11 signaling [38]. We observed an increase in the STAT3 target gene, *Socs3*, highlighting the successful activation of the signaling pathway (Fig. S5C). These results indicate that the TGFβ1 mediated EMT process, which is associated with solid MO morphology and elevated *s100a4*, is not mediated by members of the IL-6 cytokine family.

### Solid organoids form high-grade allograft tumors with increased s100a4 expression

**Fig. 6:**
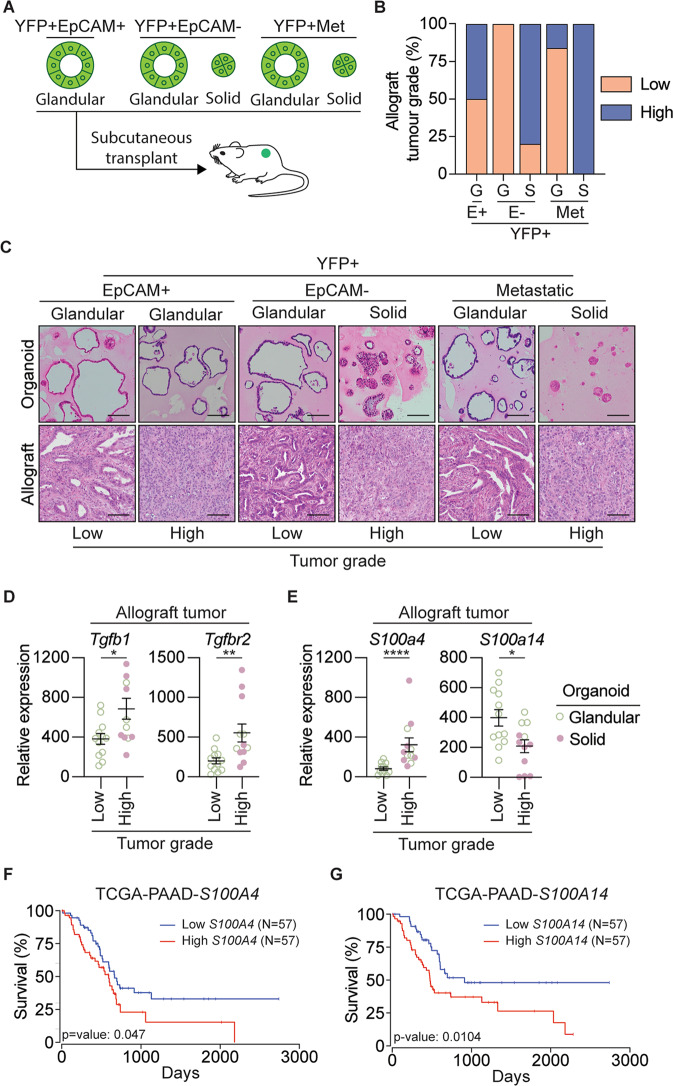
Organoid-derived allografts resemble original tumors and exhibit an EMT phenotype. **A** Schematic representation of the subcutaneous transplant of organoids into gender matched wild-type C57BL/6 mice to generate allograft lines. **B** Quantification of the number of low-grade (orange) and high-grade (blue) allograft tumors, generated from YFP + EpCAM + (E+), YFP + EpCAM- (E-) primary tumor organoids and YFP + secondary tumor organoids. Organoids are grouped as glandular (G) and solid (S) morphologies. **C** Representative H&E images of the tumor organoid, and corresponding allograft generated from YFP + EpCAM + (E + ), YFP + EpCAM- (E-) primary tumor organoids and YFP + secondary tumor organoids. Organoids are grouped as glandular (G) and solid (S) morphologies. **D**, **E** mRNA expression of *Tgfb1*, *Tgfbr2* (**D**), and the S100 family proteins, S*100a4*, *S100a14* (**E**), in low-grade (*N* = 4 tumors) and high-grade (*N* = 4 tumors) allograft tumors derived from YFP + EpCAM+ MO, YFP + EpCAM- MO, and YFP + secondary tumor MO. Each dot represents an individual tumor. Data is relative to *Gapdh*, presented as mean + /− SEM. *p < 0.05, **p < 0.01, Mann–Whitney test. **F**–**G** Kaplan–Meier plots of overall survival for PDAC patients based on high (top 33%) and low mRNA expression (bottom 33%) of *S100A4* and *S100A14* obtained from the TCGA-PAAD dataset. Plots and *p*-value were determined using the OncoLnc web tool [64].

Since we observed in our own patient cohorts that high levels of serum TGFβ1 in PDAC patients increased with the stage of the tumor (Fig. S6A), we further explored the relationship between TGFβ1, organoid morphology, and high-grade tumors by establishing MO allograft models. To this end, YFP + EpCAM+ primary tumors, YFP + EpCAM- primary tumors, and YFP + secondary tumor organoids were engrafted subcutaneously into C57B/l6 mice (Fig. 6A). We found that 100% of organoids with a solid morphology were engrafted, whereas glandular MOs had variable engraftment success and latency rates (Fig. S6B, C).

Retrospective histopathological analysis of the resulting allograft tumors indicated that nearly all solid organoids formed high-grade tumors, whereas a mixture of low-and high-grade tumors was derived from glandular organoids (Fig. 6B, C). High-grade allograft tumors exhibited classic features of EMT, including loss of EpCAM expression and increased vimentin expression (Fig. S6D). We observed that some of the glandular YFP + EpCAM- organoids re-expressed EpCAM in the allograft tumors, suggesting that MET occurred following transplantation of these organoid sub-lines (Fig. S6D). We observed higher expression of both TGFβ1 and its cognate receptor in high-grade allograft tumors (Fig. 6D). These observations corresponded with the increased expression of *S100a4* in high-grade allograft tumors, whereas *S100a14* expression was decreased (Fig. 6E).

Taken together, we have revealed that organoids with a solid morphology have a partial EMT phenotype, which can be augmented by TGFβ1 and transitioned to a complete EMT phenotype. We also showed that solid organoids form allografts, which are high-grade tumors with high expression of both TGFβ1 and *s100a4*. Our findings link TGFβ1 with changes in the expression of *S100a4* and *S100a14*, which are individually associated with poor survival in patients with PDAC (Fig. 6F, G), consistent with both proteins being elevated in tumor organoids (Fig. 2E) or patient tumors (Fig. S4B). Our results suggest that S100A4 may be an important biomarker for predicting EMT state, disease progression, and survival.

## Discussion

The convergence of tumor intrinsic and microenvironment signals that promote the transition through cancer cell EMT states, and the reversal of these processes, inevitably impacts tumor progression [39]. Given the highly aggressive nature of PDAC, the identification of biomarkers that could predict advanced disease would be clinically useful.

We used a murine model of PDAC, enabled by oncogenic recombination in epithelial cells [5]. We observed that more than half of the primary tumors displayed EMT features characterized by the gain of expression of mesenchymal markers and loss of expression of the epithelial marker EpCAM. Our study builds on the recent technical advances realized through the generation of a murine organoid biobank from primary and secondary tumors [40–42], by including a YFP reporter allele that allowed us to isolate pure epithelial cells and generate matched organoids from secondary tumors present in multiple organs, including the lung [42]. Moreover, the addition of FACS gating strategies using EpCAM as a reliable EMT lineage marker [22, 43] permitted the generation of partial EMT sublines, providing an opportunity to better understand the spectrum of partial EMT programs, which has previously been technically challenging.

The emergence of different morphologies within our organoid biobank permitted phenotyping of intrinsic cellular behaviors in a way that was not possible with monolayer cell lines [11]. Similar to previous reports on patient-derived PDAC organoids [17, 44–48], we showed that solid murine organoids are often derived from high-grade tumors. Our proteomic analysis revealed that the morphology of PDAC organoids correlates with the continuum of EMT states, which has been previously reported for mammary organoids [49, 50]. We found that within organoids isolated from both primary and secondary tumors, glandular organoids were often in an epithelial state, whereas solid organoids often underwent partial EMT. Although solid morphology was a feature of acinar organoids in previously published work [51, 52], no enrichment for acinar signatures were detected in our solid organoid proteomics dataset, ruling out an acinar origin for these samples (Fig. S4A). We also found that partial EMT organoids give rise to different EMT spectra along a previously described trajectory of the epithelial, EMT-hybrid, and mesenchymal states [22]. Interestingly, following serial passaging and allograft generation, a subset of solid organoids with a partial EMT phenotype underwent MET, characterized by reversion to a glandular phenotype and re-expression of EpCAM. This phenomenon is not uncommon, and has previously been reported ex vivo, following the culture of partial EMT cell lines [11] and in vivo following the migration of cells from primary tumors to distant organs [9, 10].

Among the proteins identified within the partial EMT state in solid organoids is the S100 protein family, which consists of 25 members with a variety of intracellular and extracellular cellular functions, including calcium homeostasis, proliferation, and apoptosis [53]. S100 proteins have also been shown to interact with cytoskeletal proteins, thereby affecting cellular morphology and migration [43, 53]. We were intrigued by the S100 proteins, as they have previously been shown to be secreted by epithelial cells in response to tissue damage or inflammatory responses [28, 54]. We found that S100a14 was increased in the organoids derived from PDAC tumors compared to the normal organoids, which is consistent with the increased expression observed in murine cancer cells and patient cancer cell lines and was linked to poor overall survival [22, 38, 55]. S100a14 has been described as a mesenchymal marker because upregulation of the transcription factor Gli1 promotes EMT and increases s100a14 expression [56]. However, using murine models [55] and human cell lines [38], we and others have shown that s100a14 is associated with epithelial and not EMT states, as its expression is lower in glandular MOs, murine tumors, and patients with low-grade tumors.

We were also interested in the increased expression of s100a4, which is associated with partial EMT in solid organoids, because s100a4 has also been associated with poor survival in PDAC [57]. S100a4 has also been shown to play a role in EMT and metastasis and is highly expressed in murine PDAC mesenchymal tumor cells, patient cell lines, and PDAC patients with high-grade tumors, suggesting a clear association with EMT [22, 38, 56]. As s100a14 is also increased in tumor cells and is associated with poor prognosis, S100A4 may be a more useful biomarker for treatment of advanced disease since high *S100A4* expression correlates with resistance to gemcitabine, the mainstay chemotherapy provided to patients with PDAC [58, 59], and EMT is known to alter the response to chemotherapy [60].

Secreted factors within the tumor microenvironment are known to influence EMT in PDAC [21]. We explored the contribution of TGFβ1 and found that recombinant TGFβ1 could transition a glandular organoid to a solid phenotype, which is associated with the induction of a classic EMT gene signature. As previously shown in PDAC patient-derived organoids [17], we observed that a branching phenotype could also be induced by recombinant TGFβ1 suggesting a transition to a complete EMT state. We were particularly intrigued by the observation that TGFβ1 could upregulate *S100a4* consistent with other studies [7, 8]. It is thought that TGFβ1 may regulate S100a4 through the EMT-TF, Zeb1, as Zeb1 overexpression also resulted in an increase in *S100A4* expression in cell lines, while *S100A14* decreased, consistent with our observations [38].

Despite being an obvious therapeutic target to prevent the transition from early stage EMT to complete EMT, there is likely limited clinical benefit following targeting of TGFβ1 in cancer due to the high probability of on-target toxicities due to the complexities of TGF signaling and function within the immune system. For this reason, we explored the role of IL-6 family cytokines in EMT, which are readily targeted therapeutically, and which we and others have shown to be induced downstream of TGFβ1 signaling [18, 19]. However, we failed to detect changes in organoid morphology following stimulation with recombinant IL-6 family cytokines nor did we detect an increase in classic EMT markers, suggesting that this cytokine family alone does not promote EMT. Moreover, despite previous reports showing that IL-11 upregulates *S100A4* and *S100A14* in human PDAC cell lines [38], IL-11Rα_1_ expression was not detected in the organoid biobank. Other studies have also shown that the treatment of lung cancer cell lines with IL-6 alone did not induce EMT, although the presence of receptor components was not confirmed [61]. We also did not observe the induction of *S100a4* following stimulation with IL-6 or LIF. However, we observed a significant induction of *S100a14* suggesting that although IL-6 and LIF may contribute to tumor features, they do not directly contribute to EMT. Thus, the therapeutic inhibition of the IL-6 cytokine family alone is unlikely to prevent EMT. However, the activation of signal transducer and activator of transcription (STAT)−3, a pro-tumorigenic transcription factor downstream of the IL-6 family of cytokines, is significantly correlated with *S100A4* expression in PDAC patient tissues [38] suggesting that cooperative induction of *S100A4* is possible. Previous reports have also suggested that synergy between ZEB1 EMT-TF and IL-6 family cytokines results in the augmentation of *S100A4* expression, which was not explored in this study [38].

Taken together, our observations suggest that both extracellular and cell-intrinsic complete EMT programs converge on S100A4 and may represent useful biomarkers for predicting disease progression and prognosis.

## Materials and methods

### Mouse strains

*Pdx*^Cre^; *Rosa*^YFP^ (CY) and *Pdx*^Cre^; *Kras*^G12V^; *p53*^R172H^; *Rosa*^YFP^ (CKPY) mice [5] were maintained on a C57BL/6 background and bred and maintained in a specific pathogen-free animal facility at WEHI. All experiments involving mice were approved by the WEHI Animal Ethics Committee (AEC approval #2019.015 and #2020.032). CKPY mice were aged between 12 and 27 weeks and were collected together with aged and sex-matched CY mice.

### Generation of organoids

FACs sorted cells from murine tissue were resuspended in >90% v/v Matrigel (Corning) and grown in murine pancreatic organoid medium (MPOM) containing advanced DMEM/F12 (Gibco) containing 10 mmol/L HEPES (Gibco), 1X GlutaMAX (Gibco), 1x penicillin/streptomycin (Gibco), 5% v/v Rspo2-Fc conditioned medium (harvested from transiently transfected FreestyleTM-293F cells, Thermo Fisher), 5% v/v Noggin-conditioned medium (harvested from Noggin-expressing 239 cells obtained from Foundation Hubrecht Organoid Technology (HUB), Hubrecht Institute, Uterecht, The Netherlands), 10 mmol/L nicotinamide, 1% v/v B-27 supplement without vitamin A, 1 mmol/L N-acetyl-L-cysteine, 100 ng/mL rh FGF-10, 50 ng/mL rh EGF, 10 nmol/L rh [Leu15]-gastrin I, and 3 µmol/L prostaglandin E2. Following passaging, the organoids were cultured in MPOM with 10 µM Y-27632 (Sigma) and 5 µM GSK-3 inhibitor (Sigma) for the first three days, followed by culturing in normal MPOM for regular maintenance, as described previously [62].

### Generation of allografts

Wild-type C57BL/6 mice were used to establish murine organoid-derived allografts. Wild-type C57BL/6 mice were bred and maintained in a specific pathogen-free animal facility at WEHI. All experiments involving mice were approved and monitored by the WEHI Animal Ethics Committee (AEC Approval #2017.033 and ##2020.032).

To generate allografts, one confluent well of organoids (approximately 50,000 cells) was resuspended in 100 µL of 50% v/v PBS/ 50% v/v Matrigel and subcutaneously injected into each flank of C57BL/6 mice (gender matched to the organoid). If no visible tumor was present 3 months after engraftment, engraftment was deemed unsuccessful and the mouse was euthanized.

### Mass spectrometry-based proteomics

Organoids were lysed in RIPA buffer containing 100 mM NaCl, 10 mM Tris-HCl, 1% (v/v) glycerol, 50 mM NaF, 2 mM EDTA, 1% (v/v) Triton X-100, 1 mM Na3VO4, complete mini protease inhibitor cocktail, and complete mini phosphatase inhibitor, and 20 µg per replicate was prepared for proteomic analysis using the USP3 protocol previously described [63] with some minor modifications. Lysates were heated at 95 **°**C for 10 min in buffer containing 1% (v/v) SDS, 100 mM Tris (pH 8), 10 mM Tris (2-carboxyethyl) phosphine (TCEP), and 40 mM 2-chloracetamide. Magnetic PureCube Carboxy agarose beads (Cube Biotech) were added to all the samples along with acetonitrile (70% v/v final concentration) and incubated at room temperature for 20 min. Samples were placed on a 96-well magnetic rack, supernatants were discarded, and beads were washed twice with 70% ethanol and once with neat ACN. ACN was completely evaporated from the tubes using a CentriVap (Labconco) before the addition of digestion buffer (50 mm Tris) containing 0.8 µg Lys-C (Wako, 129–02541) and 0.8 µg Trypsin-gold (Promega, V5280). Enzymatic digestion was performed with agitation (400 rpm) for 1 h at 37 **°**C. Following digestion, the samples were transferred to pre-equilibrated C18 StageTips for sample clean-up. The eluates were lyophilized to dryness using CentriVap (Labconco) before being reconstituted in 60 µL of 0.1% FA/2% ACN ready for mass spectrometry analysis.

For DDA analysis, peptides (2 µL) were separated by reverse-phase chromatography on a C_18_ fused silica column packed into an emitter tip (IonOpticks) using a nano-flow HPLC (M-class, Waters). HPLC was coupled to a timsTOF Pro (Bruker) equipped with a CaptiveSpray source. Peptides were loaded directly onto the column at a constant flow rate of 400 nL/min with buffer A (99.9% v/v Milli-Q water, 0.1% v/v FA) and eluted with a 90-min linear gradient from 2 to 34% buffer B (99.9% v/v ACN, 0.1% v/v FA). The timsTOF Pro was operated in PASEF mode using Compass Hystar 5.1. Settings were as follows: Mass Range 100 to 1700 m/z, 1/K0 Start 0.6 V·s/cm^2^ End 1.6 V·s/cm^2^, Ramp time 110.1 ms, Lock Duty Cycle to 100%, Capillary Voltage 1600V, Dry Gas 3 l/min, Dry Temp 180 °C, PASEF settings: 10 MS/MS scans (total cycle time 1.27 sec), charge range 0-5, active exclusion for 0.4 min, Scheduling Target intensity 10000, Intensity threshold 2500, CID collision energy 42 eV.

## Supplementary information

Supplementary Material

Supplementary Table 2

Reproducibility Checklist

Author Contribution Form

## Data Availability

Mass spectrometry proteomics data were deposited in the ProteomeXchange Consortium via the PRIDE partner repository with the dataset identifier PXD030992.
