# Correction to: S100 family proteins are linked to organoid morphology and EMT in pancreatic cancer

**DOI:** 10.1038/s41418-023-01151-y

**Published:** 2023-03-22

**Authors:** Ronnie Ren Jie Low, Ka Yee Fung, Hugh Gao, Adele Preaudet, Laura F. Dagley, Jumana Yousef, Belinda Lee, Samantha J. Emery-Corbin, Paul M. Nguyen, Rune H. Larsen, Nadia J. Kershaw, Antony W. Burgess, Peter Gibbs, Frédéric Hollande, Michael D. W. Griffin, Sean M. Grimmond, Tracy L. Putoczki

**Affiliations:** 1 The Walter and Eliza Hall Institute of Medical Research, Parkville, VIC 3052 Australia; 2 Department of Medical Biology, The University of Melbourne, Parkville, VIC 3052 Australia; 3 University of Melbourne Centre for Cancer Research, Victorian Comprehensive Cancer Centre, Parkville, VIC 3000 Australia; 4 Department of Clinical Pathology, University of Melbourne, Parkville, VIC 3000 Australia; 5 Centre for Cancer Research, Hudson Institute of Medical Research, Clayton, VIC 3168 Australia; 6 Department of Molecular and Translational Science, Monash University, Clayton, VIC 3800 Australia; 7 Department of Biochemistry and Pharmacology, Bio21 Molecular Science and Biotechnology Institute, University of Melbourne, Parkville, VIC 3000 Australia

**Keywords:** Cancer models, Cancer microenvironment

Correction to: *Cell Death & Differentiation* 10.1038/s41418-023-01126-z, published online 24 February 2023

The original version of this article contained an error in the author list. During the revision process, Dr. Paul M. Nguyen contributed to the design and assisted with the experiment presented in Supplemental Figure 4g/h. Paul’s name was omitted from the manuscript in error when it was re-submitted. The original article has been corrected.

