# Supplementary material for: S100 family proteins are linked to organoid morphology and EMT in pancreatic cancer
Source: Cell Death Differ. 2023 Feb 24;30(5):1155–65. doi: 10.1038/s41418-023-01126-z (PMC10154348; doi:10.1038/s41418-023-01126-z)
Supplement: Supplementary file 1 — Supplementary Material [file 41418_2023_1126_MOESM1_ESM.pdf]

Supplementary figures

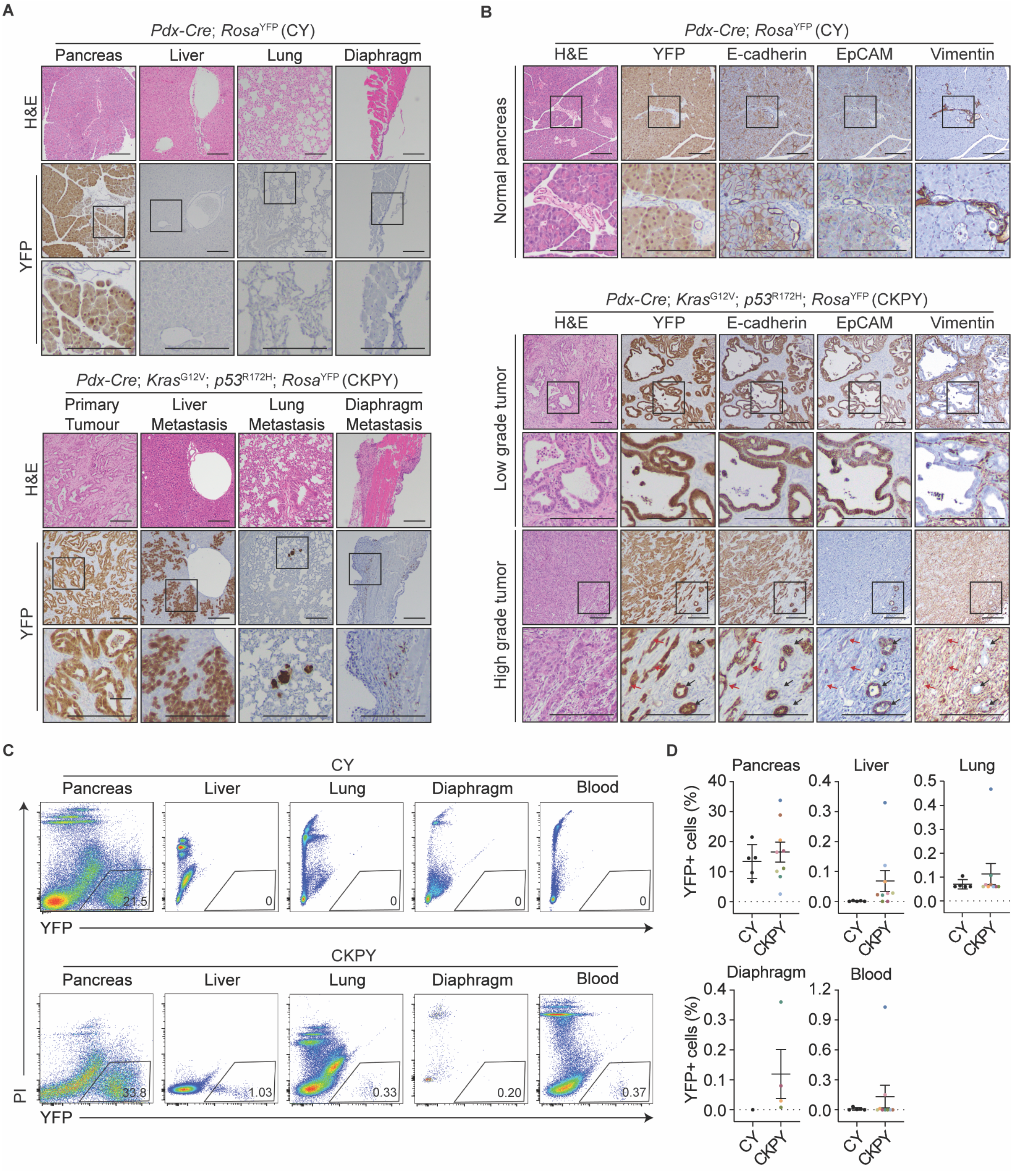

Supplementary Fig. S1. Identification and isolation of YFP+ cells from secondary tumors

(A) Representative images of the pancreas, liver, lung and diaphragm of CY mice (N=5) and CKPY (N=9) mice. Adjacent images of H&E and YFP immunohistochemsitry are shown. Scale bar = 200 μm.

(B) Representative images of normal pancreas from CY mice (top) and primary tumors from CKPY mice, separated by low grade tumor (middle) and high grade tumor (bottom). Adjacent H&E and YFP, E-cadherin, EpCAM and Vimentin stained sections are shown. YFP+EpCAM+ (black arrow) and YFP+EpCAM- (red arrow) cells are present in the high grade tumor. Scale bar = 200 μm.

(C) FACS gating strategy for YFP+ cell sorting from the pancreas, liver, lung, diaphragm and blood

harvested from CY mice (N=5) and CKPY mice (N=9).

(D) Percentage of live YFP+ cells present in the indicated organs or blood. Each dot represents an individual mouse. Data is presented as mean +/- SEM.

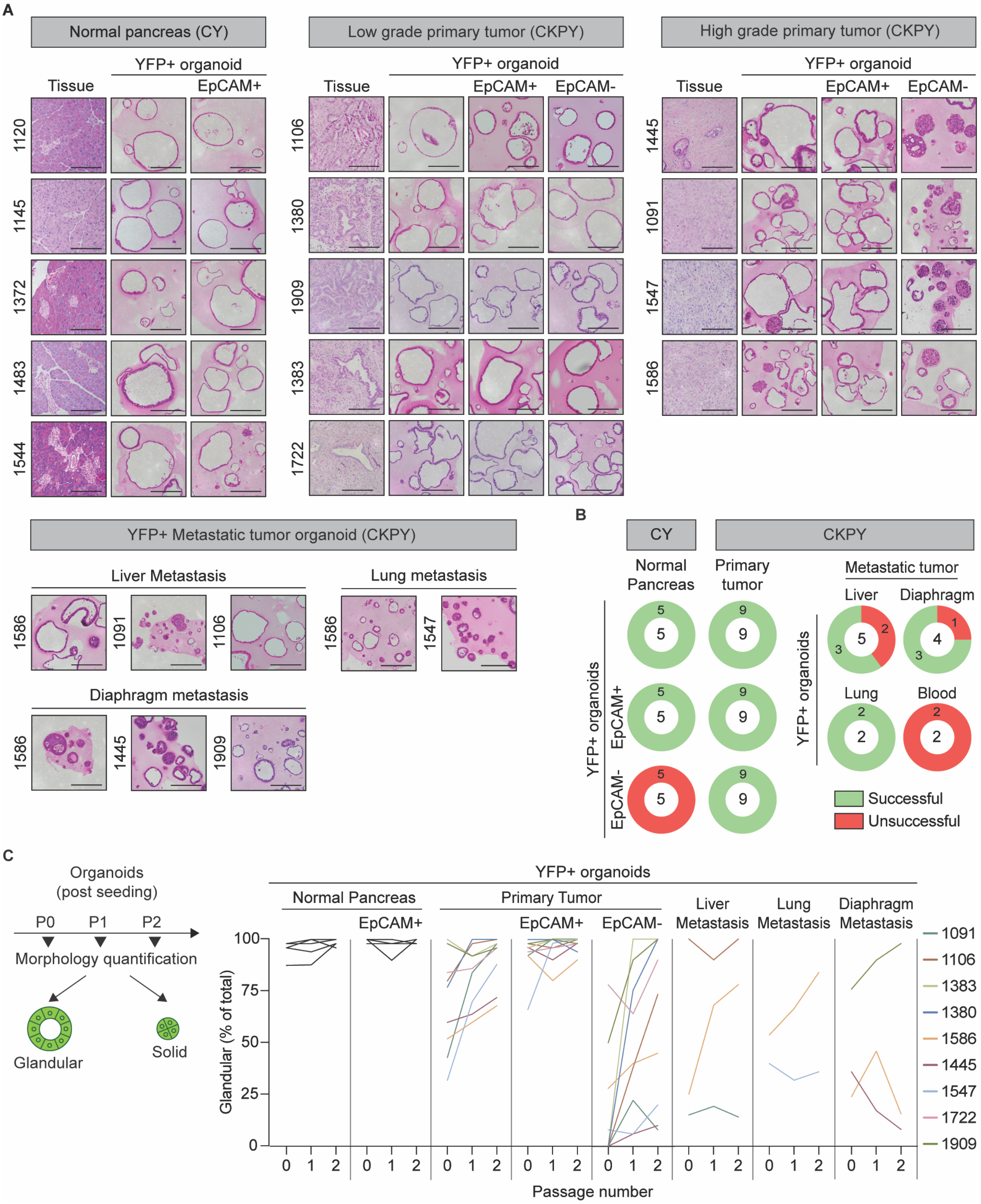

Supplementary Fig. S2. Successful growth of murine organoids from multiple tissues

(A) Representative H&E images of the primary tissue and corresponding normal pancreatic organoids generated from CY mice (N=5), or tumor organoids generated from CKPY mice (N=5 low grade, N= 4 high grade). Scale bar = 100μm.

(B) Number of organoid lines generated from live YFP+ cells sorted from the indicated organs of CY mice (N=5) or CKPY mice (N=9). Center of the plot indicates total number of organs with sorted YFP+ cells for organoid generation. Scale bar = 100μm.

(C) Quantification of the stability of organoid morphology following serial passaging (P) post seeding. Presented as the percent of glandular organoids present within the culture following P0, P1 P2 for normal (N=5), primary tumor (N=9) and metastatic tumor organoids (N=8). Data is presented as relative to total number of organoids within each well. Individual organoid lines are indicated.

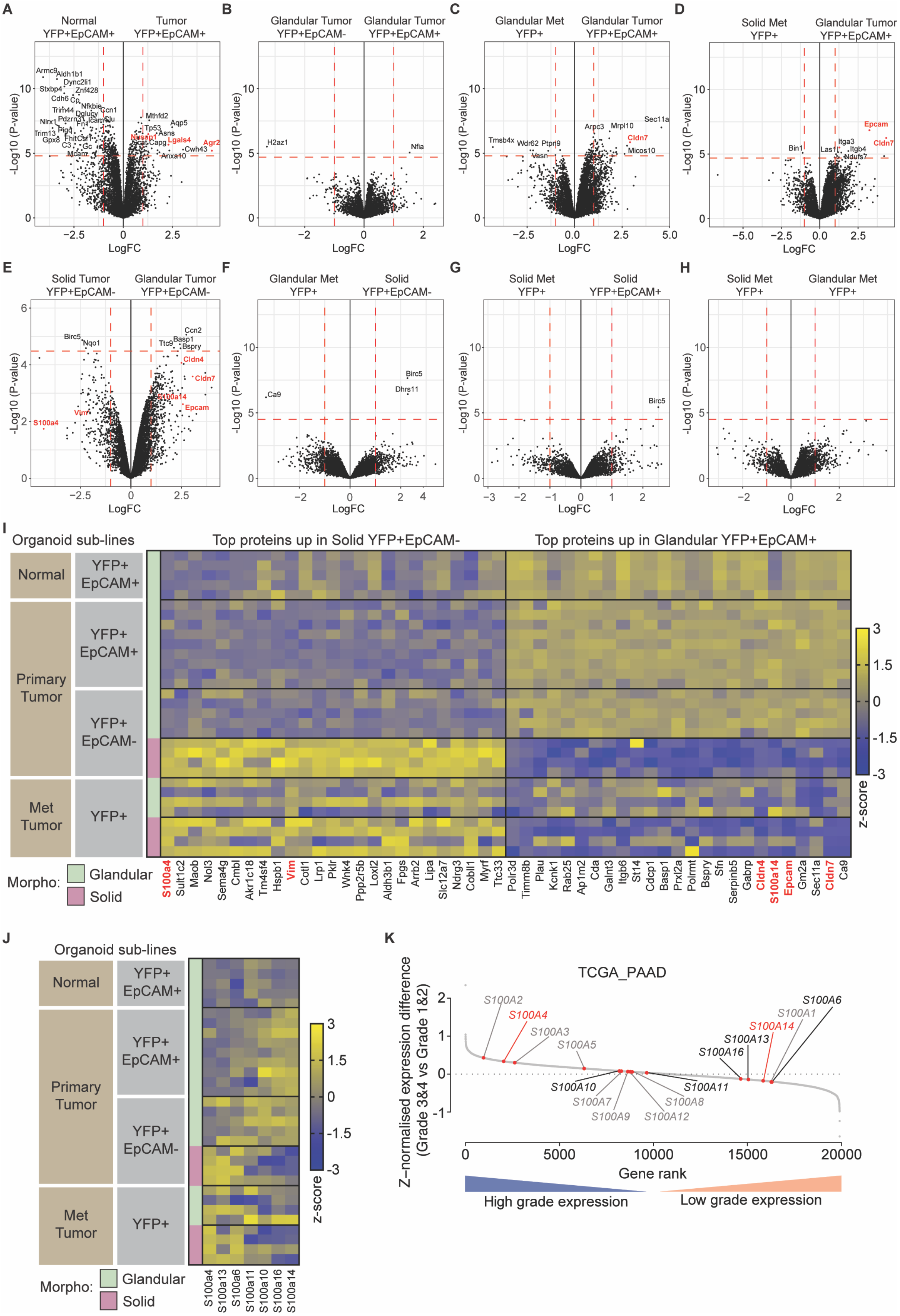

Supplementary Fig. S3. Proteomics analysis reveals different expression profile in the murine organoid sub-lines

(A-H) Volcano plot illustrating the log_2 protein ratios in whole cell lysates of organoids, comparing glandular YFP+EpCAM+ tumor organoid with normal YFP+EpCAM+ organoid (A); glandular YFP+EpCAM- tumor organoid (B); glandular YFP+ metastatic organoid (C); and solid YFP+ metastatic organoid (D); YFP+EpCAM- tumor organoids (E); metastatic organoids (F-H). Proteins were deemed differentially regulated if the log2 fold change in protein expression was ≥ 1-fold and exhibited an adjusted *p*-value ≤ 0.05.

(I-J) Heat-map representation of the top 25 differentially expressed proteins between glandular YFP+EpCAM+ tumor organoid and solid YFP+EpCAM- tumor organoid (I); S100a family proteins (J) in all the organoid lines utilized for quantitative proteomics analysis including normal YFP+EpCAM+ (N=5), tumor YFP+EpCAM+ (N=9), tumor YFP+EpCAM- (N=9) and YFP+ metastatic (N=8) tumor organoids. Organoids are grouped as glandular (G) and solid (S) morphologies.

(K) Expression of S100A family members in patient tumors ranked by the mean expression difference in grade 1/2 tumor v.s. grade 3/4 tumor from high purity tumor[1] from the TCGA-PAAD dataset. S100 family members detected in proteomics (black arrow), protein of interest (red) and other S100 family members (grey).

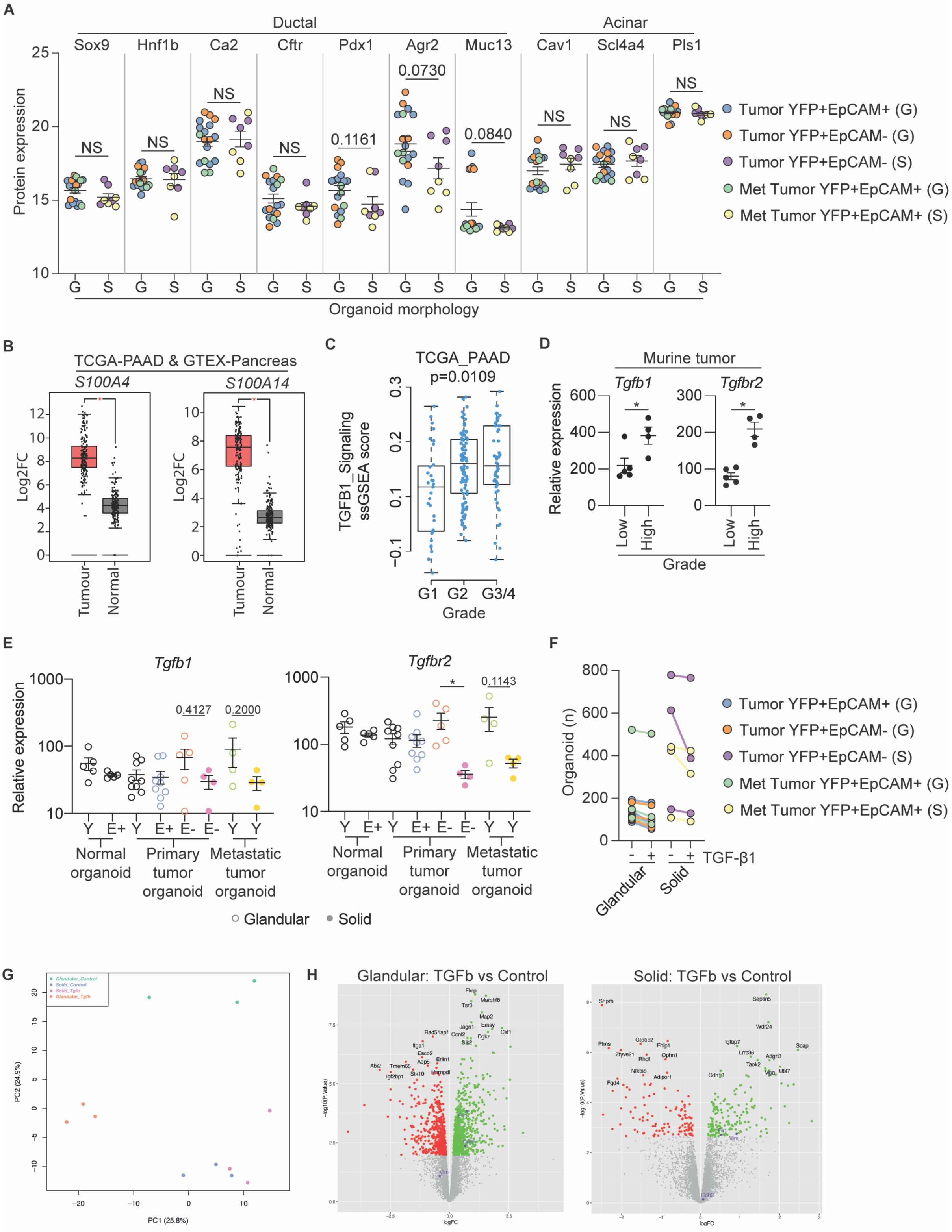

Supplementary Fig. S4. Murine organoids express *Tgfb1* and Tgfbr2

(A) Log_2 protein expression levels from organoid proteomics dataset comparing glandular (G) and solid (S) organoids. Each dot represents an individual organoid, presented as mean +/- SEM. NS: not significant, unpaired t-test presented.

(B) RNA expression analysis of *S100A4* and *S100A14* in human PDAC (TCGA-PAAD, n = 179) and normal pancreas (GTEX-pancreas, n = 171) from the GEPIA tool[2].

(C) GSEA plot evaluating the TGF-beta signaling signature (HALLMARK_TGF_BETA_SIGNALING)[3] between grade 1, grade 2 and grade 3/ 4 patients tumor from the TCGA-PAAD dataset.
(D) mRNA expression levels of *Tgfb1* and *Tgfbr2* in the original murine tumor. Each dot represents an individual tumor. Each dot represents an individual mouse. Data is relative to *Gapdh*, presented as mean +/- SEM. . *p<0.05, Mann-Whitney test.
(E) mRNA expression of *Tgfb1* and *Tgfbr2* in YFP+ (Y), YFP+EpCAM+ (E+) and YFP+EpCAM- (E-) organoids. Each dot represents an individual organoid. Data is relative to *Gapdh*, presented as mean +/- SEM. *p<0.05, Mann-Whitney test.
(F) There was no difference in organoid number between solid and glandular organoid after treatment with TGFβ1. Data is presented as number of organoids formed in TGFβ1-treated condition over vehicle control.
(G) Principle component analysis plot of 6,354 of the most variable proteins across organoids lines, including solid + TGFβ (N=3), untreated solid (N=3), glandular + TGFβ (N=3) and untreated glandular (N=3) organoids. The plot shows the separation of samples based on differential principal components (PCs). Glandular (G) and solid (S) organoid lines +/- TGFβ are indicated.
(H) Volcano plots illustrating the $\log_2$ protein rations in whole cell lysates of organoids, comparing TGFb vs control in glandular and solid tumor organoids. Proteins were deemed differentially regulated in the log2 fold change in protein expression was >1-fold and exhibited an adjusted p-value of <0.05 (proteins colored in red and green).

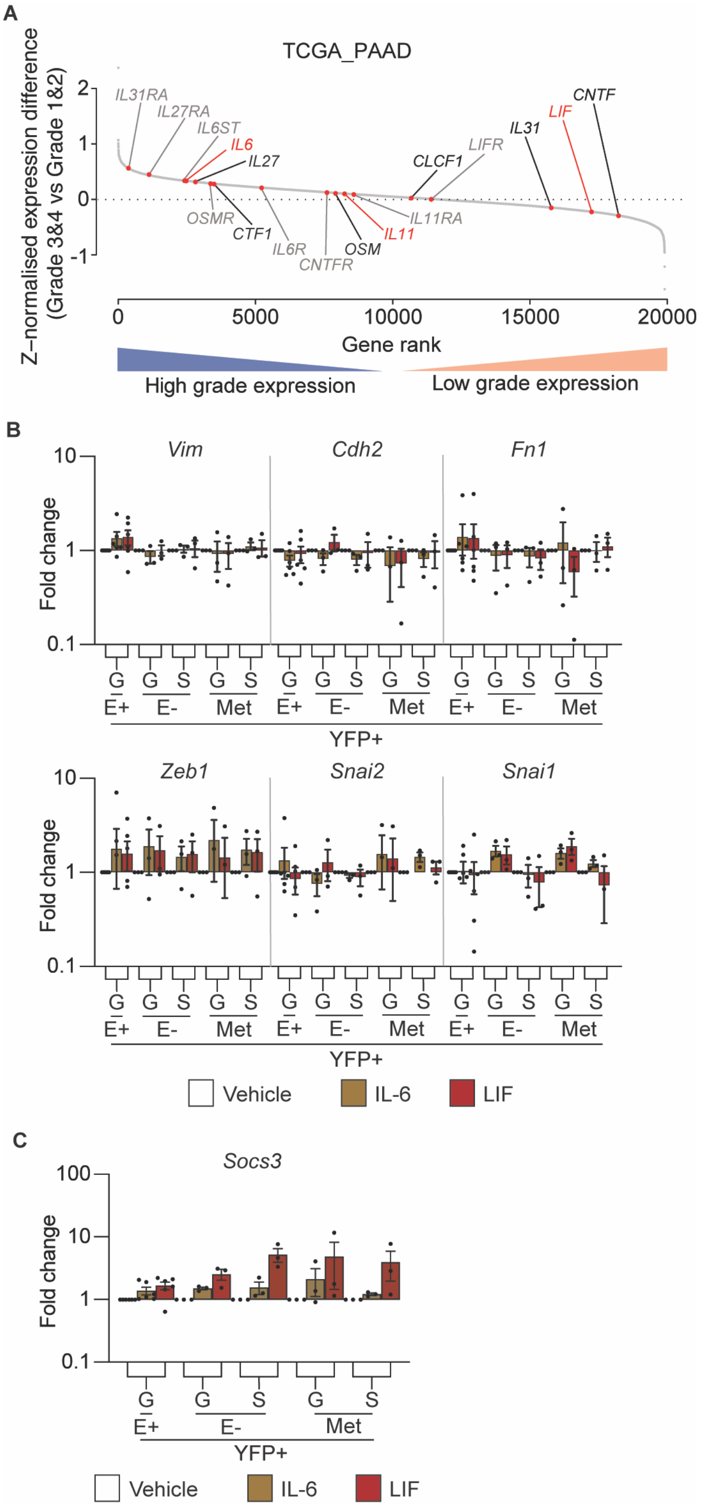

Supplementary Fig. S5. IL-6 or LIF, do not alter tumor organoid morphology or mesenchymal signature

(A) Expression of IL-6 family members in patients tumor ranked by the mean expression difference in grade 1/2 tumor v.s. grade 3/4 tumor from all tumors from the TCGA-PAAD dataset. Cytokines (black arrow), cytokines of interest (red) and cytokine receptors (grey).

(B-C) mRNA expression levels of mesenchymal markers, *Vim*, *Cdh2*, *Fn1*, *Snai1*, *Snai2*, *Zeb1* (B); and the IL-6 cytokine family target gene, *Socs3* (C), in YFP+EpCAM+ (E+), YFP+EpCAM- (E-) primary

tumor organoids and YFP+ metastatic organoids following the addition of the indicated cytokine (IL-6 brown, LIF red). Each dot represents an individual organoid. Organoids are grouped as glandular (G) and solid (S) morphologies. Data includes 3 biological replicates and is presented as log10 fold change relative to the vehicle (white) control, mean +/- SEM. paired t-test.

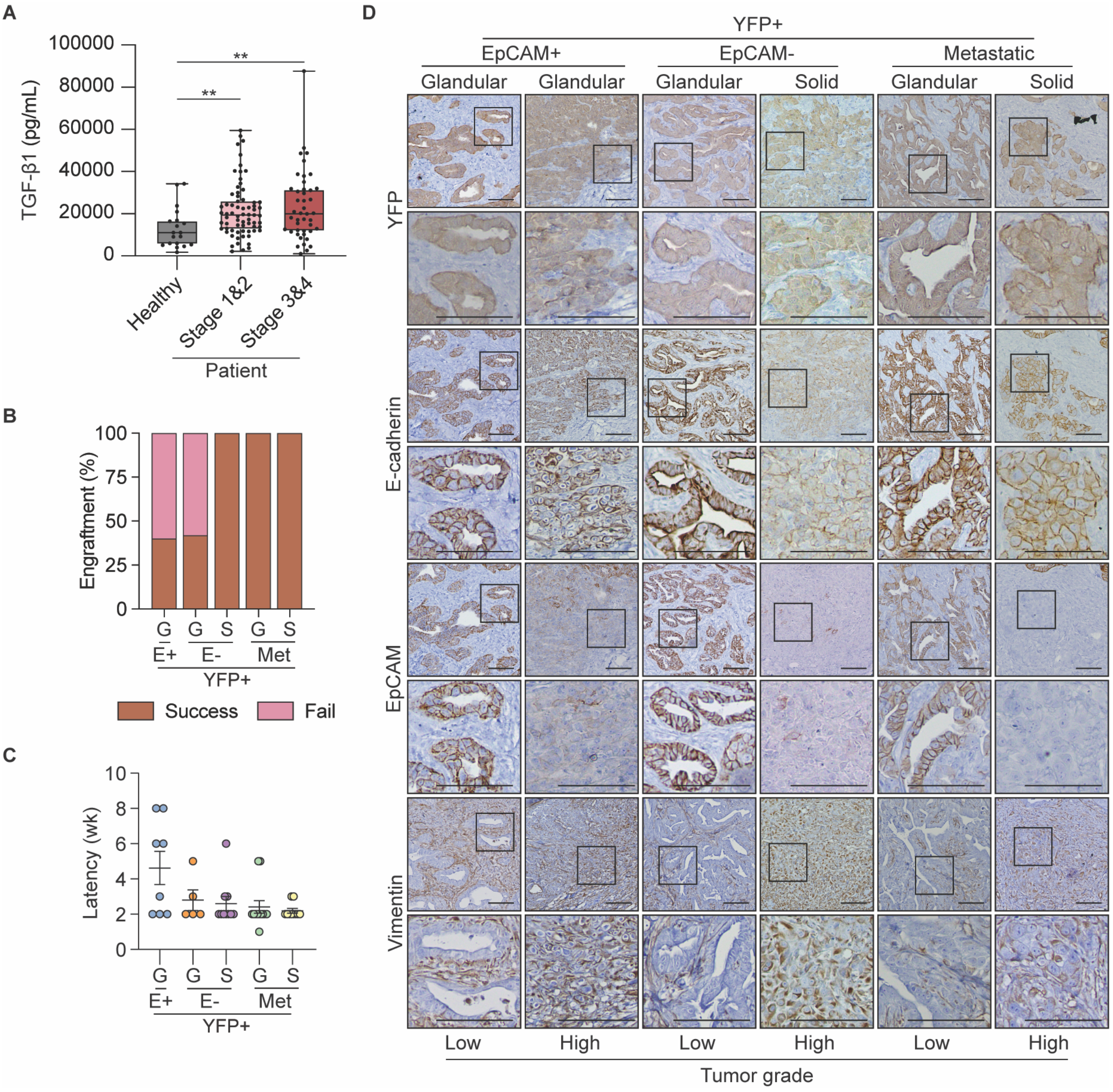

Supplementary Fig. S6. Generation of a murine allograft biobank with different histological features

(A) Serum TGFβ1 in healthy individuals (n = 19) compared to PDAC patients grouped by stage 1/2 (n = 69) and stage 3/4 (n = 45). Data is presented as box and whisker plot. *p<0.05, *p<0.01, Mann-Whitney test.

(B-C) Successful generation of allografts from YFP+EpCAM+ (E+), YFP+EpCAM- (E-) primary tumor organoids and YFP+ metastatic organoids. Organoids are grouped as glandular (G) and solid (S) morphologies. Each bar graph indicates the overall number of allografts generated by subcutaneous transplantation (N= 9 mice per organoid line). (C)Tumor latency (time to palpation) is also shown.

(D) Representative immunohistochemical staining of allografts generated from YFP+EpCAM+ (E+), YFP+EpCAM- (E-) primary tumor organoids and YFP+ metastatic organoids for YFP, E-Cadherin, and EpCAM. Organoids are grouped as glandular (G) and solid (S) morphologies.
Scale bar = 200µm.

Supplementary Table S1: Details of mice utilized for organoid generation

Genotype	Mouse #	Gender	Age (days)	Age (weeks)	Organoids Generated				
					Pancreas	Liver	Lung	Diaphragm	Blood
Pdx^{Cre}; *Rosa*^{YFP}	1210	M	122	17.4	Yes	N/D	N/D	N/A	N/U
Pdx^{Cre}; *Rosa*^{YFP}	1145	F	121	17.3	Yes	N/D	N/D	N/A	N/D
Pdx^{Cre}; *Rosa*^{YFP}	1372	F	112	16	Yes	N/D	N/D	N/A	N/D
Pdx^{Cre}; *Rosa*^{YFP}	1483	M	84	12	Yes	N/D	N/D	N/A	N/D
Pdx^{Cre}; *Rosa*^{YFP}	1544	M	140	20	Yes	N/D	N/D	N/D	N/D
Pdx^{Cre}; *Kras*^{G12V}; *p53*^{R172H}	1091	M	121	17.3	Yes	Yes	N/D	N/A	N/D
Pdx^{Cre}; *Kras*^{G12V}; *p53*^{R172H}	1106	F	119	17	Yes	Yes	N/D	N/A	N/D
Pdx^{Cre}; *Kras*^{G12V}; *p53*^{R172H}	1383	F	105	15	Yes	N/D	N/D	N/A	N/D
Pdx^{Cre}; *Kras*^{G12V}; *p53*^{R172H}	1380	M	86	12.3	Yes	N/D	N/D	N/A	N/D
Pdx^{Cre}; *Kras*^{G12V}; *p53*^{R172H}	1586	M	108	15.4	Yes	N/D	N/D	Yes	N/D
Pdx^{Cre}; *Kras*^{G12V}; *p53*^{R172H}	1445	M	164	23.4	Yes	No	Yes	N/D	N/D
Pdx^{Cre}; *Kras*^{G12V}; *p53*^{R172H}	1547	F	152	21.7	Yes	Yes	Yes	Yes	N/D
Pdx^{Cre}; *Kras*^{G12V}; *p53*^{R172H}	1722	M	190	27.1	Yes	No	N/D	No	N/D
Pdx^{Cre}; *Kras*^{G12V}; *p53*^{R172H}	1909	F	116	16.6	Yes	N/D	N/D	Yes	N/D

N/D: YFP+ cells not detected; N/A: No organ collected; N/U: YFP+ cells detectable, but not utilised for organoids.

Supplementary Table S2: Differentially expressed proteins in murine organoids

Supplementary methods

Fluorescence-Activated Cell Sorting

Pancreas, liver and lung were digested in media containing 240units/mL nystatin, 5µg/mL amphotericin B, 1% w/v FBS , 0.125 mg/mL collagenase, 0.125 mg/mL dispase II, 0.1 mg/mL DNAse I, 1x advanced DMEM/F12, 1x penicillin/streptomycin, 1x GlutaMAX, 10mM HEPES for 30 min at 37 °C. The digested samples were passed through a 70µm strainer and washed with FACS buffer (PBS with 3% v/v FBS and 1% v/v EDTA). Pancreas, liver, lung and blood samples were incubated in red blood cell lysis buffer (ThermoFisher Scientific), washed and stained with FITC-fluorochrome conjugated EpCAM antibody (Abcam, 1:1000) at 4 °C for 20 min. Propidium iodide (PI, Thermo Fisher Scientific; 1:10,000) was added to the samples prior to FACS analysis. After sample preparation, FACSAria (BD Biosciences) sorting was performed.

Histology

Murine tissues were fixed in 10% v/v formalin overnight, whereas organoids were fixed in 10% v/v formalin containing 0.5% v/v glutaraldehyde (Sigma) for 45 min, embedded in paraffin, and sectioned at a thickness of 4 µm. Hematoxylin and eosin (H&E) staining was performed using standard procedures.

Immunohistochemistry

Unstained paraffin sections were dewaxed, rehydrated, and heat-induced antigen retrieval was performed by incubating the slides in Tris-EDTA–Tween 20 buffer (10 mM Tris base, 1 mM EDTA, 0.05% v/v Tween 20, pH 9.0, pH 9) or citrate buffer (10 mM, pH 6, Thermo Fisher Scientific) as required for each primary antibody against GFP (used for YFP, Abcam, AV6673, 1:100), E-cadherin (R&D Systems, AF748, 1:100), EpCAM (CST, 14452s, 1:100), or vimentin (ABCAM, ab92547, 1:300). To block endogenous peroxidases, the slides were incubated in 3% v/v hydrogen peroxide (Biolab) for 20 min, followed by washing in ddH2O and TBST. The sections were incubated with blocking buffer (TBST with 5% v/v normal goat serum) in a humidified chamber for 1 h at RT. After blocking, the sections were incubated with the desired primary antibody diluted in blocking buffer in a humidified chamber overnight at 4°C. The slides were then washed and incubated with the appropriate HRP-conjugated secondary antibody (anti-goat, Agilent, Cat#P0449 or anti-rabbit , Agilent, Cat#P0448, 1:1000) diluted in blocking buffer for 1 h at RT.

To visualize staining, the slides were incubated in 3, 3'-diaminobenzidine (DAB, Agilent), counterstained with hematoxylin (Abcam), dehydrated, and mounted with Histomount (Life Technologies).

Imaging

Brightfield images of the organoids, histology, and histochemical slides were taken using an Olympus CX23 upright light microscope (Olympus) with an Olympus DP22 camera using CellSens Entry (Olympus) software. The organoid morphology of either glandular solid was manually quantified using ImageJ software.

Additional mass spectrometry-based proteomics

For DIA analysis, peptides (1 µL) were separated by reverse-phase chromatography on a C_{18} fused silica column (inner diameter 75 µm, OD 360 µm × 15 cm length, 1.6 µm C18 beads) packed into an emitter tip (IonOpticks) using a custom nano-flow HPLC system (Thermo Ultimate 300 RSLC Nano-LC, PAL systems CTC autosampler). The HPLC was coupled to a timsTOF Pro (Bruker) equipped with a CaptiveSpray source. Peptides were loaded directly onto the column at a constant flow rate of 400 nL/min with buffer A (99.9% Milli-Q water, 0.1% FA) and eluted with a 30-min linear gradient from 2 to 34% buffer B (90% ACN, 0.1% FA). The timsTOF Pro (Bruker) was operated in diaPASEF mode using Compass Hystar 5.1. The settings on the TIMS analyzer were as follows: Lock Duty Cycle to 100% with equal accumulation and ramp times of 100 ms, and 1/K0 Start 0.6 V.·/cm^2 End 1.6 V·s/cm^2, Capillary Voltage 1400V, Dry Gas 3 l/min, Dry Temp 180°C. The DIA methods were set up using the instrument firmware (timsTOF control 2.0.18.0) for data-independent isolation of multiple precursor windows within a single TIMS scan. The method included two windows in each diaPASEF scan, with window placement overlapping the diagonal scan line for doubly and triply charged peptides in the m/z – ion mobility plane across 16 × 25 m/z precursor isolation windows (resulting in 32 windows) defined from *m/z* 400 to 1,200, with 1 Da overlap, and CID collision energy ramped stepwise from 20 eV at 0.8 V·s/cm^2 to 59eV at 1.3 V·s/cm^2.

Data processing and statistical analysis for mass spectrometry-based proteomics

Raw DDA data files were analyzed by Fragpipe (v15.0) using a protein sequence database of reviewed murine proteins, accessed January 2021 from UniProt. The database contains 34278 entries including decoys that were generated and appended to the original using MSFragger (v3.2). Tryptic cleavage specificity was applied, allowing for two missed cleavages along with fixed carbamidomethyl cysteine, variable methionine oxidation, and N-terminal acetylation modifications. The peptide length and mass ranges were set to 7-50 residues and 500-5000 Da, respectively. The precursor mass error was set to -20-20 ppm and the fragment mass error was set to 10 ppm with mass calibration and parameter optimization. Peptide spectrum matches were filtered using PeptideProphet and ProteinProphet in Philosopher (v3.4.13) to obtain 1% PSM and 1% FDR, respectively. The peptides and proteins were quantified using IonQuant (v1.5.5), with m/z, retention time, and ion mobility tolerances set to 10 ppm, 0.4 minutes, 0.05 1/k0, respectively. Protein quant analysis was performed using the MaxLFQ algorithm, and proteins required a minimum of two peptides to be quantified.

DIA data were analysed using DIA-NN 1.8[4] in library-free mode. diaPASEF d. files were searched against reviewed sequences from mouse Uniprot Reference Proteome (downloaded November 2022) with the following settings: trypsin specificity, peptide length of 7-30 residues, cysteine

carbidomethylation as a fixed modification, variable modifications set to n-terminal protein acetylation and oxidation of methionine, the maximum number of missed cleavages at 2. Mass accuracy was set to 10 ppm for both MS1 and MS2 spectra and match between runs (MBR) enabled, and filtering outputs set at a precursor q-value < 1%.

For the DDA dataset, data processing and analysis were performed using the R software (version 4.0.4). Only proteins quantified in at least 50% of the replicates under at least one condition were retained. The protein intensities were log_2-transformed. Missing values were imputed using the missing-not-at-random (MNAR) method implemented in the msImpute R-package(v.1.2.0). The data were normalized using RUVSeq[5]. The optimum k value used to remove unwanted variations was determined based on the PCA, RLE, and p-value distribution plots. The R package DEqMS (v. 1.9.0) was used for differential analysis. For the DIA dataset, data processing and analysis were performed using R (version 4.2.1). Proteins without any proteotypic precursors or with q-value greater than 0.01 or identified by a single peptide were removed. Further filtration step was done, where proteins identified in 66% or more of samples at least in one group were kept. Total of 6,354 proteins were included in the analysis. Protein intensities were log2-transformed and normalised using RUVIIIC (v. 1.0.19). Invariant proteins in all conditions (P-value > 0.5), with coefficient of variation (CV%) < 2% , were chosen as negative controls for RUVIIIC normalisation. Missing values were imputed by applying Barycenter approach for Missing Not At Random (v2-MNAR) method implemented in msImpute package (v. 1.7.0). Differential analysis was performed using limma (v. 3.52.4). A protein was determined to be significantly differentially expressed if the false discovery rate (FDR)-adjusted p value was ≤ 0.05. A protein was determined to be significantly differentially expressed if the false discovery rate (FDR) was ≤ 5% after Benjamini–Hochberg (H-B) correction. The R packages ggplot2 (v. 3.3.3) and superheat (v. 0.1.0) were used to visualize the results.

EMT signature genes were obtained from Simeonov *et a*l (2020). Protein expression of EMT signature genes, which were detected, was summarized using the ssGSEA algorithm as implemented in the GSVA package for R. The statistical significance of differences in the ssGSEA signature score between groups was calculated using Welch's t-test, and Benjamini-Hochberg correction was performed, as implemented in the R statistical package.

RNA isolation, cDNA synthesis and qPCR

Tissue samples were homogenized in TRIzol (Ambion) using a Tissue Lyser II (Qiagen). One milliliter of chloroform (Merck Millipore) was added to each tube, mixed gently, and centrifuged for 15 min at 3,000 rpm at 4°C. The upper layer was transferred into a 1.5 mL tube (Eppendorf) containing 350 µL of 100% v/v isopropanol. RNA extraction was performed using a Qiagen RNA Mini Kit (Qiagen), according to the manufacturer's protocol. Murine organoids were grown until confluence and released from Matrigel by incubation in 500 µL of organoid harvesting solution (Gibco) at 4 °C with gentle rocking for 45 min. The samples were washed, and RNA extraction was performed using a Qiagen RNA minikit

(Qiagen) according to the manufacturer's protocol. cDNA was generated using a high-fidelity cDNA synthesis kit (Applied Biosystems), according to the manufacturer's instructions.

The qRT-PCR was performed on a Viia 7 real-time PCR system with the following steps: initial denaturation for 10 min at 95 °C, denaturation for 20 s at 94 °C, annealing for 15 s at 60 °C and extension for 15 s at 72 °C for 40 cycles using gene-specific Taqman probes (Applied Biosystems) for *Cdh2* (Mm01162497_m1), *Fn1* (Mm01256744_m1), *Gapdh* (Mm99999915_g1), *Il11* (Mm00434162_m1), *Il11ra1* (Mm01223545_m1), *Il6* (Mm00446190_m1), *Il6ra* (Mm01211445_m1), *Il6st* (Mm00439665_m1), *Lif* (Mm00434762_g1), *Lifr* (Mm00442942_m1), *S100a4* (Mm00803372_g1), *S100a14* (Mm04206817_g1), *Snai1* (Mm00441533_g1), *Snai2* (Mm00441531_m1), *Socs3* (Mm00545913_s1), *Tgfb1* (Mm01178820_m1), *Tgfbr2* (Mm03024091_m1), *Vim* (Mm01333430_m1), *Zeb1* (Mm00495564_m1). A relative comparative threshold (CT) method was used for qRT-PCR analysis using Microsoft Excel (Microsoft). ΔCT values were calculated by subtracting the CT of the gene of interest from that of the housekeeping genes. ΔCT was then calculated to determine the individual normalized values (2-ΔCT). The results for organoids are presented as fold change relative to matched YFP+ organoids. Results for cytokine stimulation are presented as fold-change relative to vehicle.

Cytokine stimulations

Organoids were plated as single cells in 2 wells of a 24-well plate for each condition. Vehicle (PBS) and recombinant cytokines, including 10 ng/mL of TGFβ1 (R&D Systems, 140-B), 100 ng/mL IL-6 (Laboratory of Michael Griffin), or 100 ng/mL LIF (Laboratory of Michael Griffin), were diluted in MPOM and added to the culture on day 0 (at the time of seeding). On day 3 post-stimulation, brightfield images of the organoids were taken for quantification and harvested for qPCR.

Patient Serum

Blood was collected from de-identified healthy or PDAC patients who had consented to a project governed by WEHI (G16/05), and serum collected using a Sarstedt tube at 400 g for 15 min for storage at -80 °C.

TGF-beta1 Quantification

The serum level of TGF-β1 was measured using a commercially available Bio-Plex Pro TGF-β Assay (Bio-Rad Laboratories Ltd., Hercules, CA, USA) on the Bio-Plex™ 200 System. The Bio-Plex™ 200 software version 5.1.1 (Bio-Rad Laboratories) was used to determine the concentration in pg/mL.

Analysis of publicly available gene expression datasets

TCGA pancreatic cancer z-distribution-transformed expression data were obtained from cbioportal.org[6] and correlated with the histopathological classifications provided with the data-set [7]. Gene expression differences between groups were ranked based on differences in mean z-scores. For gene set enrichment analysis of TCGA gene expression data, the ssGSEA algorithm was used, as implemented

in the GSVA package. TGFB1_Signalling ssGSEA was generated based on a 54 markers defined in HALLMARK_TGF_BETA_SIGNALING from the Molecular Signatures Database (MSigDB) hallmark gene set collection[3]. Patient survival analysis on the TCGA pancreatic cancer dataset was performed using a publicly available web tool http://www.oncolnc.org/,[8]. Gene expression comparison between human PDAC from TCGA and normal pancreas from GTEX was performed using the Gene Expression Profiling Interactive Analysis (GEPIA) webtool http://gepia.cancer-pku.cn/ [2].

General statistical analysis

Organoid morphology quantification of either glandular or solid were manually quantified using ImageJ. All results were plotted using the GraphPad Prism software. The statistical tests performed are described in the legend of figures. A result was considered significant if the P-value was less than 0.05, and has been indicated in the graphs as * P-value <0.05, ** P-value <0.01, *** P-value <0.001, and *** P-value <0.0001.

References

1. Raphael BJ, Hruban RH, Aguirre AJ, Moffitt RA, Yeh JJ, Stewart C, *et al.* Integrated Genomic Characterization of Pancreatic Ductal Adenocarcinoma. *Cancer Cell* 2017, **32**(2)**:** 185-203.e113.

2. Tang Z, Li C, Kang B, Gao G, Li C, Zhang Z. GEPIA: a web server for cancer and normal gene expression profiling and interactive analyses. *Nucleic Acids Research* 2017, **45**(W1)**:** W98-W102.

3. Liberzon A, Birger C, Thorvaldsdóttir H, Ghandi M, Mesirov JP, Tamayo P. The Molecular Signatures Database (MSigDB) hallmark gene set collection. *Cell Syst* 2015, **1**(6)**:** 417-425.

4. Demichev V, Szyrwiel L, Yu F, Teo GC, Rosenberger G, Niewienda A, *et al.* dia-PASEF data analysis using FragPipe and DIA-NN for deep proteomics of low sample amounts. *Nat Commun* 2022, **13**(1)**:** 3944.

5. Risso D, Ngai J, Speed TP, Dudoit S. Normalization of RNA-seq data using factor analysis of control genes or samples. *Nat Biotechnol* 2014, **32**(9)**:** 896-902.

6. Gao J, Aksoy BA, Dogrusoz U, Dresdner G, Gross B, Sumer SO, *et al.* Integrative analysis of complex cancer genomics and clinical profiles using the cBioPortal. *Sci Signal* 2013, **6**(269)**:** pl1.

7. Integrated Genomic Characterization of Pancreatic Ductal Adenocarcinoma. *Cancer cell* 2017, **32**(2)**:** 185-203.e113.

8. Anaya J. OncoLnc: linking TCGA survival data to mRNAs, miRNAs, and lncRNAs. *PeerJ Computer Science* 2016, **2:** e67.
