# Supplementary material for: S100 family proteins are linked to organoid morphology and EMT in pancreatic cancer
Source: Cell Death Differ. 2023 Feb 24;30(5):1155–65. doi: 10.1038/s41418-023-01126-z (PMC10154348; doi:10.1038/s41418-023-01126-z)
Supplement: Supplementary file 4 — Author Contribution Form [file 41418_2023_1126_MOESM4_ESM.pdf]

DECLARATION OF CONTRIBUTIONS TO ARTICLE

ADMC

Manuscript Number: CDD-22-0869

Journal Name: *Cell Death & Differentiation* (the 'Journal')

Proposed Title of the Contribution: authorship contributions (the 'Contribution)

Author(s): The indicated authors. (the 'Authors')

For all *CDD* articles, each person named as an author in the published version must be able to show he or she has contributed substantially to the article.

Authorship credit should be based on 1) substantial contributions to conception and design, acquisition of data, or analysis and interpretation of data; 2) drafting the article or revising it critically for important intellectual content; and 3) final approval of the version to be published. Authors should meet conditions 1, 2 and 3.

Any person who cannot be shown to have made a substantial contribution to the article cannot be listed as an author in the final version. The name of any person who is deemed to have made a minor contribution can, however, appear in the Acknowledgments section of the article.

Please complete the table below to indicate the contributions of all named authors to the manuscript.

Author Full Name:	Specification of Contribution to the Manuscript:
Ronnie Ren Jie Low	Conceived of the study, designed the experiments, and wrote the manuscript. Performed all MO experiments
Ka Yee Fung	Contributed to in vivo experiments. Performed the serum analyses.
Hugh Gao	Analyzed the public transcriptomic and proteomic datasets
Adele Preaudet	Contributed to in vivo experiments.
Laura F. Dagley, Jumana Yousef, Rune H. Larsen	Performed proteomic analysis
Belinda Lee	Performed the serum analyses.
Nadia J. Kershaw, Antony W. Burgess	Contributed critical reagents and intellectual input.
Peter Gibbs	Provided human samples
Frédéric Hollande	Supervised experiments and provided intellectual support.
Michael D.W. Griffin	Contributed critical reagents and intellectual input. Supervised experiments and provided intellectual support
Sean M. Grimmond	Supervised experiments and provided intellectual support.
Tracy L. Putoczki	Conceived of the study, designed the experiments, supervised the experiments, provided intellectual input and wrote the manuscript

Please complete the table below to indicate the contributions of all named authors to the figures.

Figure 1:

RRJL generated the data and prepared all panels. KFY assist with generating the data. NJK and AWB contributed critical reagents and intellectual input. FH, MDWG, SMG and TLP supervised experiments and provided intellectual support. TLP and RRJL prepared the figure.

Figure 2:

RRJL generated the data and prepared all panels. HG, LFD, JY and RHL generated the data. FH, MDWG, SMG and TLP supervised experiments and provided intellectual support. TLP and RRJL prepared the figure.

Figure 3:

RRJL generated the data and prepared all panels. FH, MDWG, SMG and TLP supervised experiments and provided intellectual support. TLP and RRJL prepared the figure.

Figure 4:

RRJL generated the data and prepared all panels. FH, MDWG, SMG and TLP supervised experiments and provided intellectual support. TLP and RRJL prepared the figure.

Figure 5:

RRJL generated the data and prepared all panels. MDWG contributed critical reagents and intellectual input. FH, SMG and TLP supervised experiments and provided intellectual support. TLP and RRJL prepared the figure.

Figure 6:

RRJL generated the data and prepared all panels. KFY, AP, BL and PG assist with generating the data. FH, MDWG, SMG and TLP supervised experiments and provided intellectual support. TLP and RRJL prepared the figure.

Signed for and on behalf of the Author(s):	Print Name:	Date:
	Tracy Putoczki	27.04.2022
